# Trends and variation in unsafe prescribing of methotrexate: a cohort study in English NHS primary care

**DOI:** 10.3399/bjgp20X710993

**Published:** 2020-06-23

**Authors:** Brian MacKenna, Helen J Curtis, Alex J Walker, Richard Croker, Seb Bacon, Ben Goldacre

**Affiliations:** EBM DataLab, Nuffield Department of Primary Care Health Sciences, University of Oxford, Oxford.; EBM DataLab, Nuffield Department of Primary Care Health Sciences, University of Oxford, Oxford.; EBM DataLab, Nuffield Department of Primary Care Health Sciences, University of Oxford, Oxford.; EBM DataLab, Nuffield Department of Primary Care Health Sciences, University of Oxford, Oxford.; EBM DataLab, Nuffield Department of Primary Care Health Sciences, University of Oxford, Oxford.; Nuffield Department of Primary Care Health Sciences, University of Oxford, Oxford.

**Keywords:** drug prescribing, epidemiology, general practice, methotrexate

## Abstract

**Background:**

Prescribing high doses of methotrexate increases the potentially fatal risk of toxicity. To minimise risk, it is recommended that only 2.5 mg tablets are used.

**Aim:**

To describe trends in GP prescribing of methotrexate over time; the harm associated with methotrexate errors at a national level; ascertain variation between practices and clinical commissioning groups (CCGs) in their implementation of the safety guidance; and map current variations at CCG and practice level.

**Design and setting:**

A retrospective cohort study of English GP prescribing data (August 2010–April 2018), and data acquired via freedom of information (FOI) requests.

**Method:**

The main outcome measures were: variation in ratio of non-adherent/adherent prescribing, geographically and over time, between practices and CCGs; and description of responses to FOI requests.

**Results:**

Of 7349 practices in England, 1689 prescribed both 2.5 mg and 10 mg tablets to individual patients in 2017, breaching national guidance. In April 2018, 697 practices (≥90th percentile) prescribed >14.3% of all methotrexate as 10 mg tablets, likewise breaching national guidance. The 66 practices at ≥99th percentile gave >52.4% of all prescribed methotrexate in the form of 10 mg tablets. The prescribing of 10 mg tablets fell during the study period, with 10 mg tablets as a proportion of all prescribed methotrexate tablets falling from 9.1% to 3.4%. Twenty-one deaths caused by methotrexate poisoning were reported from 1993–2017 in England and Wales.

**Conclusion:**

The prevalence of unsafe methotrexate prescribing has reduced but remains common, with substantial variation between practices and CCGs. The authors recommend investment in better strategies around implementation. As 21 deaths that occurred from 1993–2017 in England and Wales were attributed to methotrexate poisoning, the coroners’ reports for these deaths should be reviewed to identify recurring themes.

## INTRODUCTION

Methotrexate is a folic acid antagonist, commonly prescribed for conditions such as rheumatoid arthritis, Crohn’s disease, severe psoriasis, and some cancers. It has a narrow therapeutic index, and high doses can result in potentially fatal adverse events, including blood disorders, liver toxicity, and shortness of breath.^1^ Due to its narrow therapeutic index, and an unusual once-weekly dosing regimen, this drug presents a particularly high risk of accidental overdose: patients may incorrectly take methotrexate on a daily basis and overdose may occur due to dispensing or prescribing errors. The *British National Formulary* (BNF) states that methotrexate should, usually, only be prescribed in a single strength of tablet, usually 2.5 mg, to reduce the risk of harm from errors.^1^

An NHS inquiry was convened in 2000, following the death of a patient who had accidentally taken a higher dose of methotrexate daily; the inquiry made 28 recommendations to minimise the future risk of harm.^2^ In 2006, the (now-defunct) National Patient Safety Agency (NPSA) (see Supplementary Box S1 for a glossary of referenced organisations) issued a national-level patient-safety alert, accompanied by a series of guides and recommendations to reduce the risk of harm to patients.^3^ Similar incidents have occurred across Europe and, because of the persistence of such issues, the European Medicines Agency (EMA) issued new guidance to prevent methotrexate dosing errors.^4^^,^^5^

Correct prescribing of methotrexate remains a key priority for the NHS; it is one of 16 targeted issues in the current NHS Never Events list.^6^ In addition, NHS Improvement has issued further guidance on Never Events to supplement the work of the NPSA — there, the correct prescribing of methotrexate is one of only 11 targeted issues.^7^ The NHS Improvement document states that:

*‘All electronic prescribing and dispensing software programmes in primary and secondary care locations must include oral methotrexate alerts and prompts.’* ^7^

Surprisingly, however, there is no mention of monitoring compliance in routine data or feeding back to practices and clinical commissioning groups (CCGs) if a breach of guidance is identified.

EBM DataLab’s OpenPrescribing service (openprescribing.net) is a publicly funded and accessible explorer for NHS primary care prescribing data; it was launched in 2015, and in 2019 had 130 000 unique users, including doctors, pharmacists, and patients. It supports complex bespoke data queries and displays numerous predefined standard measures for safety, cost, and effectiveness for every GP practice in England. OpenPrescribing has a standard measure for methotrexate prescribing,^8^ and shows the proportion prescribed in potentially dangerous 10 mg tablet doses. The authors noted that a substantial number of practices and CCGs are commonly in breach of best-practice guidance around methotrexate and, therefore, set out to:

- describe the long-term trends in GP prescribing of methotrexate over time;
- ascertain variation between practices and CCGs in their implementation of the safety guidance;
- map current variations at CCG and practice level; and
- describe the harm associated with methotrexate errors at a national level.

**Table:** How this fits in

In the UK, it is recommended that, when prescribing oral methotrexate tablets, only 2.5 mg tablets should be used; this is to minimise the risk of accidental overdose, which can be fatal. This study shows that breaches of this guidance are common, and vary widely between practices: 9.5% (*n* = 697) of all practices (*n* = 7349) give ≥14.3% of their methotrexate as 10 mg tablets; and 1% of practices (*n* = 66) give ≥52.4% as 10 mg tablets. Twenty-one deaths caused by methotrexate poisoning have been reported in England and Wales from 1993 until 2017. Anyone can view monthly data on all general practices breaching national methotrexate safety guidance (openprescribing.net/measure/methotrexate), supporting audit and review of current practice.

## METHOD

### Study design

Prescribing practice was analysed by conducting a retrospective cohort study using prescribing data from all English NHS general practices and CCGs. Harm associated with methotrexate was assessed by requesting data via freedom of information (FOI) requests to the Office for National Statistics (ONS) and NHS Resolution.

### Data sources

Data were extracted from the OpenPrescribing database. This imports openly accessible prescribing data from the large, monthly files published by the NHS Business Services Authority, which contain data on cost and items prescribed for each month for every typical general practice and CCG in England, dating back to mid-2010.^9^ The authors excluded a small number of settings such as walk-in centres, which typically do not issue repeat prescriptions for medicines, and where no data on EHR usage is available. The monthly prescribing datasets contain one row for each different medication and dose in each prescribing organisation in NHS primary care in England, describing the number of items (that is, prescriptions issued) and the total cost. These data are sourced from community pharmacy claims data and, therefore, contain all items that were dispensed. All available prescribing data were extracted for institutions identified as typical general practices; all other organisations, such as prisons or specialist community clinics, were excluded using NHS Digital organisation data.^10^ The number of patients registered at each practice was obtained from NHS Digital data.^11^

In addition, aggregated patient-level data were requested^12^ from the NHS Business Services Authority to ascertain where co-prescribing of both methotrexate 2.5 mg and 10 mg tablets occurred for an individual patient. FOI requests were also sent to ONS and NHS Resolution; in brief, requested data related to deaths from methotrexate (ONS)^13^ and associated legal claims and costs (NHS Resolution). These requests and responses can be viewed on Figshare.^14^

### Methotrexate prescribing

Data were extracted on all prescriptions dispensed between August 2010 and April 2018 — the latest data available when the analysis commenced — for methotrexate of any form, using BNF codes starting with 0801030P (injections, ampoules, and pre-filled pens) and 1001030U0 (tablets, liquids, and pre-filled pens). Data on liquids were excluded because of the low volume of prescribing. CCG and practice-level deciles were calculated at each month for the proportion of total methotrexate tablets prescribed as 10 mg tablets; these were then plotted on a time-series chart. Additionally, the authors analysed data supplied from the NHS Business Services Authority on prescribing of both 2.5 mg and 10 mg tablets to individual patients.

### Geographical variation at CCG level across England

Choropleth maps of the overall proportion of methotrexate tablets prescribed as 10 mg tablets between May 2017 and April 2018 were created for each CCG in England.

### Factors associated with prescribing of methotrexate 10 mg tablets

Factors associated with a high proportion (>10%) of 10 mg methotrexate tablet prescribing were examined using a mixed-effects logistic regression model. Variables from data available on individual CCGs and practices were then selected from publicly available data that have previously been shown to be associated with variation in prescribing. These variables were:

- Index of Multiple Deprivation score;
- Quality and Outcomes Framework (QOF) score;
- a composite prescribing score (determined by taking the mean percentile of the existing OpenPrescribing measures) (Supplementary Box S2);^15^
- the primary electronic health record (EHR) system used in the practice;
- whether a practice was single-handed;
- the urban/rural nature of the practice;
- proportion of patients aged >65 years;
- proportion of patients aged <18 years; and
- proportion of patients with a long-term health condition.^16^

The CCG impact was explored as a random effect to estimate the influence of CCG membership on individual practices within their organisation. Continuous variables were categorised a priori into quintiles in order to allow for non-linearity of effects and to enhance the intelligibility of results.

The outcome used was a binary variable of whether a practice had >10% of methotrexate prescriptions issued with 10 mg tablets. This threshold was selected as the majority of practices had no 10 mg methotrexate prescribing, but the authors did not want to include practices that only very occasionally prescribed 10 mg doses. The model was used to calculate odds ratios (ORs) and 95% confidence intervals (CIs) for each of the fixed-effect variables, as well as an *R* ^2^ value (along with the statistical significance level) to describe the degree of variance associated with CCG membership.

### Harms associated with methotrexate errors at a national level

Responses to FOI requests were aggregated and summarised.

### Software and reproducibility

Data management was performed using Python 3 and Google BigQuery, with analysis carried out using Stata (version 14.2) and Python 3. Data, as well as all code for data management and analysis, are archived online and available for re-use free of charge, including in Jupyter Notebook.^17^

### Patient and public involvement

OpenPrescribing receives a large volume of user feedback from professionals, patients, and the public. This feedback was used to refine and prioritise EBM DataLab’s informatics tools and research activities. Patients were not formally involved in developing this specific study design.

## RESULTS

### Methotrexate prescribing

**Figure 1.**
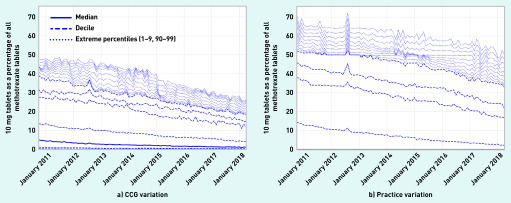
**Prescribing of 10 mg tablets as a proportion of all methotrexate tablets prescribed across CCGs and practices in England, October 2010–April 2018. CCG = clinical commissioning group.**

Methotrexate 10 mg tablets represented 3.4% of all methotrexate tablet prescribing in April 2018; this reduced from 9.1% in August 2010. Figure 1 shows the trends and variation in prescribing of 10 mg tablets as a proportion of all methotrexate tablets across England’s practices and CCGs between October 2010 and April 2018. Although the general trend is downwards, there is still extensive variation. In April 2018, most practices prescribed no methotrexate 10 mg tablets (median 0.0%). However, 697 NHS GP practices in England (≥90th percentile) prescribed ≥14.3% of all methotrexate as 10 mg tablets, in breach of BNF guidance. The 66 practices at ≥99th percentile gave ≥52.4% of all prescribed methotrexate in the form of 10 mg tablets.

Co-prescribing of methotrexate 2.5 mg and 10 mg tablets in individual patients was further investigated by submitting an FOI request to the NHS Business Services Authority for the number of individual patients receiving both 10 mg and 2.5 mg tablets in the same prescription. For reasons of information governance, low patient numbers (between *n* = 1 and *n* = 4) were suppressed at source. In total, 1689 of 7349 (23.0%) practices co-prescribed both methotrexate 2.5 mg and 10 mg tablets to individual patients, against existing safety guidance. In total, 197 (2.7%) practices prescribed mixed strengths for in excess of five of their individual patients, involving 1826 patients in total.

### Geographical variation at CCG level across England

**Figure 2.**
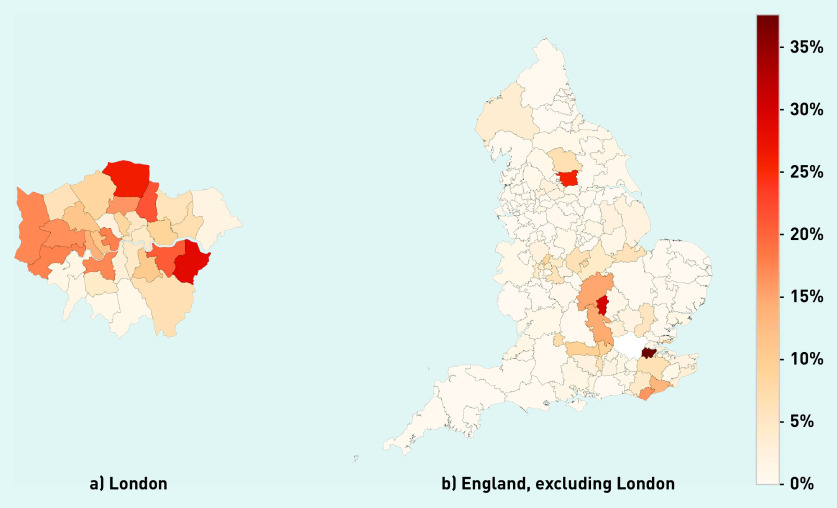
**Variation between CCGs of 10 mg items as a proportion of all methotrexate tablets in a) London and b) England, excluding London, May 2017–April 2018. CCG = clinical commissioning group.**

Figure 2 shows the variation in prescribing of 10 mg tablets as a proportion of all tablets over 12 months across England (median 1.1%, range 0.0–37.6%) and London (median 8.6%, range 0.9–28.6%). The 10 CCGs with highest prescribing of 10 mg methotrexate in breach of existing BNF safety guidance are listed in Table 1, along with the proportion of prescriptions that breached guidance.

**Table 1.** Top 10 CCGs prescribing 10 mg items as a percentage of all methotrexate tablets prescribed, May 2017–April 2018

**CCG**	**Measure of prescribing of 10 mg items as a proportion of all methotrexate tablets, %**
NHS Dartford, Gravesham, and Swanley CCG	37.6
NHS Milton Keynes CCG	29.8
NHS Bexley CCG	28.6
NHS Enfield CCG	26.2
NHS Leeds CCG	25.6
NHS Waltham Forest CCG	21.4
NHS Greenwich CCG	20.8
NHS Central London (Westminster) CCG	17.9
NHS Hounslow CCG	17.7
NHS Hillingdon CCG	17.3

CCG = clinical commissioning group.

### Factors associated with prescribing of methotrexate 10 mg

The practice factors associated with prescribing a high proportion (>10%) of methotrexate 10 mg tablets were modelled (Table 2).

**Table 2.** Absolute proportion of 10 mg methotrexate prescribing, stratified by various practice factors, along with odds ratios from a univariable and multivariable logistic regression model.

**Variable**	**Practices with >10% 10 mg methotrexate prescribing, %**	**Univariable logistic regression**		**Multivariable logistic regression**	
	
		**OR**	**95% CI**	**OR**	**95% CI**
**Patients aged >65 years, %^a^**					
0–10.9	27.2	Reference	Reference		
10.9–15.5	19.3	0.64	0.54 to 0.76	0.76	0.59 to 0.99
15.5–18.9	13.6	0.42	0.35 to 0.51	0.63	0.45 to 0.89
18.9–22.5	8.1	0.23	0.19 to 0.29	0.36	0.24 to 0.55
22.5–92.2	5.4	0.15	0.12 to 0.20	0.27	0.16 to 0.45

**Patients aged <18 years, %^a^**					
0–17.8	11.6	Reference	Reference		
17.8–19.6	10.7	0.92	0.73 to 1.16	0.85	0.62 to 1.17
19.6–21.2	12.9	1.13	0.90 to 1.41	0.69	0.49 to 0.95
21.2–23.6	17.6	1.63	1.32 to 2.01	0.71	0.51 to 0.99
23.6–53.6	20.8	2.01	1.63 to 2.46	0.68	0.48 to 0.96

**Patients with a long-term health condition, %^a^**					
16.5–47.0	23.6	Reference	Reference		
47.0–51.5	18.4	0.73	0.61 to 0.87	0.88	0.69 to 1.12
51.5–55.3	12.7	0.47	0.39 to 0.58	0.87	0.66 to 1.15
55.3–59.7	10.6	0.38	0.31 to 0.47	0.81	0.60 to 1.09
59.7–96.0	8.3	0.29	0.24 to 0.37	0.78	0.56 to 1.09

**Single-handed practice**					
No	14.6	Reference	Reference		
Yes	15.9	1.11	0.87 to 1.42	1.21	0.87 to 1.70

**Urban/rural setting**					
Urban, with major conurbation	24.1	Reference	Reference		
Urban, with minor conurbation	1.3	0.04	0.02 to 0.11	0.12	0.02 to 0.62
Urban, with city and town	10.2	0.36	0.30 to 0.42	0.18	0.10 to 0.35
Urban, with significant rural	9.3	0.32	0.25 to 0.41	0.13	0.06 to 0.26
Largely rural	5.3	0.18	0.13 to 0.25	0.15	0.07 to 0.31
Mainly rural	5.0	0.17	0.11 to 0.24	0.15	0.07 to 0.33

**Index of Multiple Deprivation quintile**					
5 (least deprivation)	12.0	Reference	Reference		
4	12.5	1.05	0.84 to 1.31	1.04	0.75 to 1.44
3	16.5	1.46	1.18 to 1.80	0.98	0.69 to 1.38
2	16.9	1.49	1.21 to 1.84	0.99	0.67 to 1.46
1 (most deprivation)	15.7	1.37	1.11 to 1.69	1.19	0.77 to 1.84

**Quality and Outcomes Framework score^a^**					
14–523	19.0	Reference	Reference		
523–541	17.3	0.89	0.74 to 1.07	0.89	0.69 to 1.14
541–550	13.8	0.68	0.56 to 0.83	0.77	0.59 to 1.00
550–557	13.1	0.64	0.52 to 0.78	0.80	0.61 to 1.04
557–559	10.0	0.47	0.38 to 0.58	0.65	0.48 to 0.88

**Composite OpenPrescribing score^a,b^**					
<38.7	15.3	Reference	Reference		
38.7–43.5	14.5	0.94	0.76 to 1.15	1.26	0.96 to 1.66
43.5–47.8	13.4	0.86	0.70 to 1.05	1.39	1.04 to 1.87
47.8–52.4	13.8	0.89	0.72 to 1.09	1.96	1.44 to 2.67
>52.4	16.4	1.09	0.89 to 1.33	3.28	2.35 to 4.57

**Computer system**					
EMIS (Egton Medical Information Systems)	14.1	Reference	Reference		
Evolution	1.7	0.11	0.01 to 0.78	1.57	0.15 to 16.58
SystmOne	13.9	0.98	0.85 to 1.13	0.97	0.70 to 1.34
Vision	29.5	2.56	2.03 to 3.24	1.68	1.07 to 2.65

a Figures are rounded.

b Lower score is indicative of higher-quality prescribing. CI = confidence interval. OR = odds ratio.

Demographic factors were associated with prescribing methotrexate 10 mg tablets. Practices with a higher proportion of patients aged >65 years were less likely to have high rates of 10 mg prescribing (multivariable OR for highest versus lowest: 0.27, 95% CI = 0.16 to 0.45), as were those with a higher proportion of patients aged <18 years (OR for highest versus lowest: 0.68, 95% CI = 0.48 to 0.96).

Similarly, practices with a high QOF score had lower rates of prescribing of 10 mg tablets (multivariate OR for highest versus lowest: 0.65, 95% CI = 0.48 to 0.88) Urban areas were especially likely to have high 10 mg tablet prescribing rates (Table 2), but it is likely that this is predominantly due to a focus of 10 mg tablet prescribing in London and Leeds (Figure 2).

Having a higher (worse) composite OpenPrescribing score was associated with a greater likelihood of high 10 mg tablet prescribing (Table 2).

Prescribing rates, largely, did not correlate with the principal EHR system a practice used, with the exception of Vision; practices using Vision were more likely to have high 10 mg tablet prescribing (OR for Vision versus EMIS: 1.68, 95% CI = 1.07 to 2.65).

The CCG to which a practice belongs (as a random effect) was significantly associated with high-dose prescribing (*P*<0.0001) and accounted for 25.5% of the variation in methotrexate 10 mg tablet prescribing.

### Harms associated with methotrexate errors at a national level

ONS data showed 21 reported deaths from 1993 until 2017, classified as a poisoning, in which methotrexate was the only drug mentioned on the death certificate; in total, there were 24 deaths due to poisoning in which methotrexate was mentioned on death certificates in England and Wales. The data reported by ONS and NHS Resolution were not sufficient to explore in any detail how methotrexate was involved in the reported death.^14^

## DISCUSSION

### Summary

At least 21 people died from methotrexate poisoning in England and Wales between 1993 and 2017. The prescribing of methotrexate 10 mg tablets remain, common, but practice is extremely variable: most practices prescribed none, but 697 NHS GP practices in England prescribed ≥14.3% of all methotrexate as 10 mg tablets; the percentage for 66 practices was ≥52.4%. Breaches of existing guidance were more common in urban practices and those with a worse composite OpenPrescribing quality score. CCG membership explained 25.5% of prescribing variation, suggesting that CCGs exert a substantial influence on clinical practice regarding methotrexate.

### Strengths and limitations

OpenPrescribing data includes all prescribing in all typical practices in England, thereby minimising the potential for obtaining a biased sample. Real prescribing and spending data, which are sourced from pharmacy claims, were used; as such, these did not need to rely on surrogate measures. This was complemented with aggregated patient-level data to identify patterns of co-prescribing, which, again, were sourced from pharmacy claims. Using primary data, rather than survey data, eliminates the possibility of recall bias. Ideally, hospital prescribing would also have been included; the authors have advocated for these data to be more widely shared but, at present, they are only available for pharmaceutical industry marketing and a limited range of unpublished analyses at NHS Improvement.

Data on deaths related to methotrexate were obtained from ONS, the most robust dataset available on drug poisonings. This dataset is comprehensive but figures are for deaths related to those registered in each calendar year, rather than those that occur in each year. In addition, a coroner’s inquest can take months, or even years, to complete.

The data on legal claims obtained from NHS Resolution have limited structure and do not give any information on the reason for the claim: deaths data may include deaths not related to primary care prescribing, for example, after prescriptions that were given in hospital or adverse reactions not caused by excessive dosing.

### Comparison with existing literature

The authors are aware of no prior work on the prevalence of breaches on methotrexate guidance; however, incomplete implementation of the important national NHS safety alert is consistent with extensive prior work showing incomplete or slow adoption for other national prescribing guidance.^18^^,^^19^ A 2008 survey of 376 members of the British Association of Dermatology reported 49 deaths of patients taking methotrexate; of these, one was caused by confusion between the 2.5 mg and 10 mg doses, and two were caused by daily rather than weekly dosing.^20^ However, these survey data rely on recall over many years of a doctor’s career unlike the present study, which used data from death certificates where poisoning related to methotrexate was mentioned.

One article, published in 2006, reported 137 patient-safety incidents related to methotrexate in England over the previous 10 years;^21^ however, these figures are likely to include a wide range of issues, including inconsistent documentation by clinicians regarding dose (such as using 2.5 mg tablets only), prescribing of concomitant folate, and use of blood tests.

National organisations in France, Canada, and Australia have also issued advice to prescribers, similar to the NHS; also similar to the findings presented here, deaths and other errors associated with methotrexate prescribing continue to occur in these countries.^22^^–^^24^

### Implications for research and practice

Previous work across several countries has resulted in a variety of suggested approaches to minimising the risks involved with methotrexate prescribing,^22^^,^^23^^,^^25^ and the EMA has recently issued further guidance.^4^^,^^5^ NHS Improvement has classed overdoses associated with methotrexate as a Never Event;^6^ however, the authors found no evidence of any action taken by any national NHS body when anomalous prescribing has been detected in a region or practice; this is concerning but may, in part, be explained by the fact that the responsibility for enacting change is unclear. The Never Event framework was initially managed by the NPSA; this body closed in 2012 and became part of NHS Improvement, which does not have any remit over primary care; this may change as NHS Improvement merges with NHS England.

Locally, the NHS has invested extensively in medicines optimisation activity, in which teams of pharmacists in every CCG monitor prescribing behaviour and advocate for change with individual clinicians. Previous work has shown that CCG membership is associated with prescribing patterns for practices,^19^^,^^26^^,^^27^ and, in the present study, a statistically significant relationship was found between the CCG to which a practice belonged and the variation in prescribing of methotrexate 10 mg tablets. It is very concerning that a number of CCGs exhibit minimal change-related activity in response to an important safety alert. In the authors’ view, there is room for substantial improvement in local staff training, alongside open data monitoring by NHS England, and appropriate action for those failing to implement change.

Practices with a higher OpenPrescribing score were also more likely to have a higher proportion of 10 mg tablets prescribed than those with a lower score. It is unlikely that methotrexate causes poor prescribing in other areas, or poor prescribing in other areas cause breaches of safety guidance when prescribing methotrexate. The authors propose that both aspects of prescribing are linked by more fundamental issues, such as individual clinicians’ skills on evidence-based medicine, or the extent to which the practice team works together to review prescribing behaviour as indicated by their own practice’s data, identify areas in which they are outliers or exhibit unusual prescribing, and take action collectively to address these issues.

The scale of breaches of existing guidance for methotrexate is clear; however, it is likely that many other safety issues exhibit similarly prevalent breaches. Given the low cost and high impact of data analyses in identifying individual practices and the scale of national problems, it is suggested that this should be a high priority for research. Although many deaths have been attributed to methotrexate, data from death certificates are scant. Accessing and reviewing the text of coroners’ reports for all deaths associated with methotrexate would establish the role the drug played in these deaths and help identify preventive strategies; although this would take time, this level of review seems consistent with the prioritisation of correct methotrexate dosing as a Never Event.

Decision-support tools and pop-ups that feature in a clinician’s EHR software at the point of care may offer an important opportunity to block unsafe prescribing; the methotrexate safety alert from the NPSA specifically highlights that EHRs should include alerts and prompts.^3^ The NHS makes significant investments in EHRs^28^ and it is imperative that their user interfaces help health professionals to prescribe safely. It was found that one of the EHR systems used in the NHS (Vision) was associated with higher prescribing of 10 mg tablets; this may reflect weaker preventive measures in this system. This finding should be investigated promptly to understand whether the design choice of the user interface in Vision increases the likelihood that a patient is prescribed 10 mg tablets.

More generally, the authors have been repeatedly blocked from researching the impact of pop-ups on prescribing, as there is no national framework or data on which pop-ups are implemented in each setting. This also means that NHS commissioners and leaders cannot routinely identify which pop-ups are implemented across the NHS. In order to realise the often-cited potential of technology to improve safety, the NHS needs better oversight of technology and the ability to ensure system providers make modifications quickly if, or when, shortcomings are identified.^29^^,^^30^

In summary, the prevalence of unsafe methotrexate prescribing has gradually reduced, but it remains common and with substantial variation between GP practices. This is unlikely to be a unique problem. The authors recommend that the NHS invests in better strategies around audit and targeted dissemination of safety information, and identifies named individuals and roles with responsibility for implementing the recommendations of safety alerts.
